# A furoviral replicase recruits host HSP70 to membranes for viral RNA replication

**DOI:** 10.1038/srep45590

**Published:** 2017-04-03

**Authors:** Jian Yang, Fen Zhang, Nian-Jun Cai, Ne Wu, Xuan Chen, Jing Li, Xiang-Feng Meng, Tong-Quan Zhu, Jian-Ping Chen, Heng-Mu Zhang

**Affiliations:** 1 State Key Laboratory Breeding Base for Zhejiang Sustain Pest and Disease Control; MOA and Zhejiang Key Laboratory of Plant Protection and Biotechnology, Institute of Virology and Biotechnology, Zhejiang Academy of Agricultural Sciences, Hangzhou 310021, China; 2 College of Chemistry and Life Science, Zhejiang Normal University, Jinhua 321004, China; 3 Zhejiang Agriculture and Forest University, Linan 311300, China; 4 Zhumadian Academy of Agriculture Sciences, Zhumadian 463000, China

## Abstract

Many host factors have been identified to be involved in viral infection. However, although furoviruses cause important diseases of cereals worldwide, no host factors have yet been identified that interact with furoviral genes or participate in the viral infection cycle. In this study, both TaHSP70 and NbHSP70 were up-regulated in Chinese wheat mosaic furovirus (CWMV)-infected plants. Their overexpression and inhibition were correlated with the accumulation of viral genomic RNAs, suggesting that the HSP70 genes could be necessary for CWMV infection. The subcellular distributions of TaHSP70 and NbHSP70 were significantly affected by CWMV infection or by infiltration of RNA1 alone. Further assays showed that the viral replicase encoded by CWMV RNA1 interacts with both TaHSP70 and NbHSP70 *in vivo* and *vitro* and that its region aa167–333 was responsible for the interaction. Subcellular assays showed that the viral replicase could recruit both TaHSP70 and NbHSP70 from the cytoplasm or nucleus to the granular aggregations or inclusion-like structures on the intracellular membrane system, suggesting that both HSP70s may be recruited into the viral replication complex (VRC) to promote furoviral replication. This is the first host factor identified to be involved in furoviral infection, which extends the list and functional scope of HSP70 chaperones.

RNA viruses are ubiquitous biotic stresses on both unicellular and multicellular organisms and include many important pathogens of humans, domestic and farm animals and of crops. Their successful infection and symptom development depends on complex molecular interactions between viral and host factors. In recent years, many host factors have been identified that interact with viral genes and are involved in viral infection cycles and symptom expression. Among these, heat shock proteins (HSPs), highly conserved and ubiquitous cytoprotective proteins, often play crucial roles in important processes,. including replication, virion assembly, and intracellular movement^1^. Based on molecular mass and sequence homology, HSPs can be classified into five major categories: HSPp100/ClpB, HSP90, 70 kDa heat shock protein (HSP70/DnaK), chaperonin (HSP60/GroEL), and small heat shock protein (sHSP).

HSP70s are central to the cellular chaperone network, and have long been recognized as among the most abundant conserved molecular chaperones in all living organisms^2^,^3^, assisting nascent protein folding in normal physiological conditions as well as under biotic and abiotic stress. In Arabidopsis, at least 14 members of the HSP70 family have been found to be unequally distributed among its five chromosomes and to have enhanced expression at particular developmental stages or to be induced by stresses^4^,^5^. Genetic evidence has illustrated the essential functions of HSP70s in embryogenesis^6^, root and shoot development, hormone signalling pathways^7^ and immune and abiotic stress responses^8^,^9^. In wheat, a major staple crop plant, quantitative PCR and proteomic analysis showed that its HSP70 genes are expressed throughout the early grain development stages^10^,^11^,^12^; and are up-regulated by the addition of CaCl_2_^13^, by heat stress^10^,^14^ and by infection with the stripe rust fungus^15^. suggesting that wheat HSP70s play important roles although their detailed functions remain largely unknown.

*Chinese wheat mosaic virus* (CWMV) is a member of the genus *Furovirus*, family *Virgaviridae*^16^. Like all known furoviruses, CWMV causes a damaging disease of cereal plants and has rigid rod-shaped particles with a bipartite single-strand positive RNA (ssRNA) genome^17^. RNA1 (7,147 nt) encodes three proteins: (1) a 153 KDa replicase protein (p153); (2) a 212 KDa protein (p212) produced by occasional read-through of the UGA termination codon of p153 to incorporate an RdRP domain; (3) a cell-to-cell movement protein (MP). RNA2 (3,564 nt) is predicted to encode four proteins: the major coat protein (CP: 19 kDa), two minor CP-related proteins (N-CP: 23 kDa; CP-RT: 84 kDa) produced by translation initiation at a non-canonical CUG start codon or occasional read-through of the UGA termination codon, respectively, and a cysteine-rich RNA silencing suppressor (CRP: 19 kDa)^17^,^18^. Recently, infective full-length cDNA clones of CWMV have been developed^19^ but so far no host factors have been identified that interact with the furoviral genes and participate in the infection cycle of CWMV, or of other furoviruses. In this study, the expression of a host HSP70 gene was found to be regulated by CWMV infection and required for replication of CWMV. Its functions during viral infection were further dissected in host plants.

## Results

### Heat shock protein 70 s (HSP70s) were regulated by, and essential for, CWMV infection in host plants

**Figure 1:**
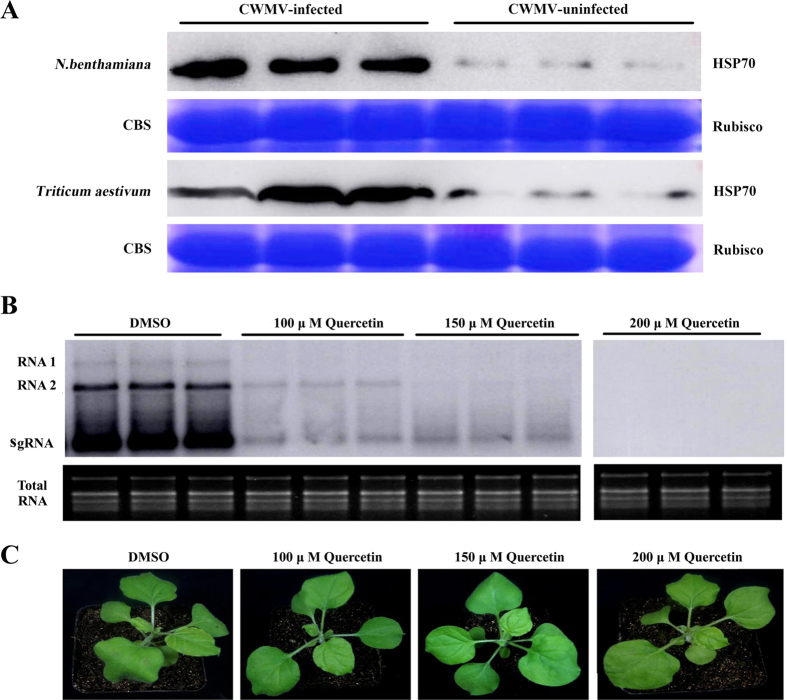
CWMV infection induced the accumulation of heat shock protein 70s (HSP70s) (**A**) and inhibition of HSP70s by quercetin treatment reduced the accumulation of CWMV RNAs in plants (**B**). (**A**) Western-blotting analysis of HSP70 in CWMV-infected *Nicotiana benthamiana* (top line) and *Triticum aestivum* (bottom line) using an anti-HSP70 antibody. Rubisco stained with Coomassie brilliant blue (CBB) was used for the loading controls. (**B**) Northern blotting assay of CWMV RNA accumulation in *N. benthamiana* plants treated with various concentrations of quercetin. Ethidium bromide (EtBr)-stained rRNAs are shown as the loading controls. Treatment with DMSO was chosen as the control. sgRNA: subgenomic RNA. (**C**) Symptoms developed at 4 dpi on quercetin- and DMSO-treated *N. benthamiana* plants.

Western blot analysis of systemically infected leaves collected from *N. benthamiana* plants 7 days post-infiltration (dpi) with infectious CWMV cDNA clones and from infected *Triticum aestivum* with typical mosaic symptoms showed that they had significantly more HSP70 than the corresponding healthy plants (Fig. 1A).

To investigate the roles of HSP70 in CWMV infection, its expression was reduced in *N. benthamiana* (Supplemental Figure S1A) by quercetin, a flavonoid chemical inhibitor specific for HSP70 expression in both single cells and whole organisms^20^. Interestingly, the accumulation of CWMV RNA in *N. benthamiana* was severely reduced when infected leaves were treated with 100 or 150 μM quercetin and no CWMV RNA was detected 4 days after treatment with 200 μM (Fig. 1B,C). These experiments suggested that HSP70s might be required for CWMV infection.

### An HSP70 from *Triticum aestivum* (TaHSP70) and its homolog in *N. benthamiana* (NbHSP70) were highly regulated by CWMV infection

**Figure 2:**
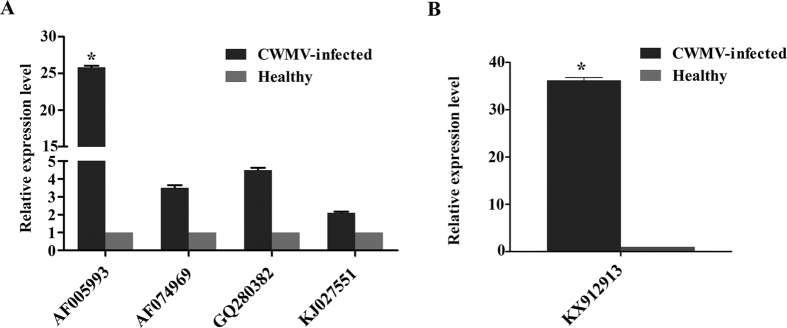
Expression levels of heat shock protein 70 (HSP70) transcripts in CWMV-infected *Triticum aestivum* (**A**) and *Nicotiana benthamiana* (**B**) plants. Experiments were repeated at least three times. Bars represent the standard errors of the means. Three sample unequal variance directional *t*-test was used to test the significance of the difference (*p < 0.05).

The initial experiments could not distinguish which member(s) of the HSP70 family were affected by CWMV infection. We therefore searched the NCBI transcriptional database of wheat and found 27 expressed sequence tags (ESTs). These ESTs were assembled into four contigs of HSP70-like genes, which were almost identical to those in previous reports, including AF005993^10^,^14^, AF074969^21^, GQ280382^15^, and KJ027551^22^. We then designed primer pairs for quantitative RT-PCR (qRT-PCR) and characterized the expression profile of these genes in *Triticum aestivum* with or without CWMV infection. The genes corresponding to AF074969, GQ280382, and KJ027551 increased 2 to 5-fold (Fig. 2A) while the gene of AF005993 increased by more than 25-fold in infected wheat plants, indicating that these HSP70 genes could be regulated to varying degrees by CWMV infection. We selected the most sensitive gene (accession number: AF005993, designated TaHSP70 in this study for convenience) for further investigation.

*N. benthamiana* is an excellent experimental host of CWMV^18^ and an efficient and reliable agro-infiltration method has been developed for viral reverse genetic assays on it^19^. To further characterize the relationship between HSP70s and CWMV infection, we then examined the expression of an ortholog of TaHSP70 from *N. benthamiana* (Genbank accession number: KX912913) which was designated NbHSP70 for convenience in this study and had 93.1% aa identity (98.3% similarity) to TaHSP70 (See Supplementary Figure S2). A quantitative assay showed that the transcriptional expression of NbHSP70 was up-regulated by more than 30-fold at 7 dpi in CWMV-infected *N. benthamiana* (Fig. 2B).

### Both NbHSP70 and TaHSP70 enhanced CWMV genomic RNA accumulation in plants

**Figure 3:**
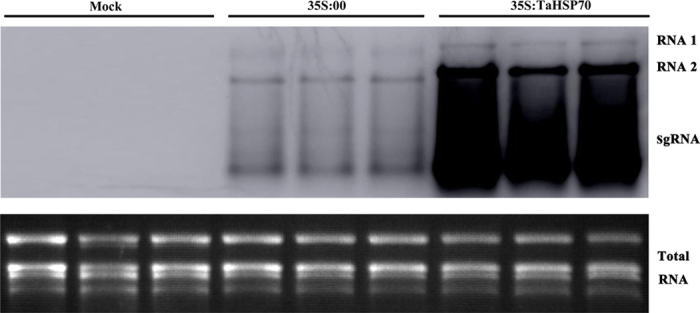
Over-expression of TaHSP70 enhanced the accumulation of CWMV RNAs in *N. benthamiana* plants. Total RNAs were extracted from the inoculated leaves 4 dpi after co-inoculation with 35S:TaHSP70 and CWMV RNAs by *Agrobacterium* infiltration. Accumulation of CWMV RNAs was analyzed by Northern blotting. Ethidium bromide (EtBr)-stained rRNAs are shown as loading controls. Inoculation with water and empty vector (35S:00) was used as the negative control. Three replicate plants were used for each treatment.

To investigate the function of HSP70, a binary vector (35S:TaHSP70) was constructed and introduced into *N. benthamiana* epidermal cells for transient overexpression of TaHSP70 (see Supplementary Figure S1B). To analyze the effect of its overexpression on CWMV infection, the binary vector was co-infiltrated with CWMV infectious clones. As controls, plants were mock-inoculated with ddH_2_O or co-infiltrated with an empty binary vector with CMV 35S promoter (35S:00) and CWMV infectious clones. Total RNAs were extracted from the inoculated or mock-inoculated leaves at 4 dpi and used for Northern blot assays. CWMV genomic RNA (gRNA) accumulated much more highly in the plants expressing TaHSP70 than in control plants (Fig. 3). Similar overexpression of NbHSP70 in *N. benthamiana* also enhanced the accumulation of CWMV genomic RNAs (data not shown).

**Figure 4:**
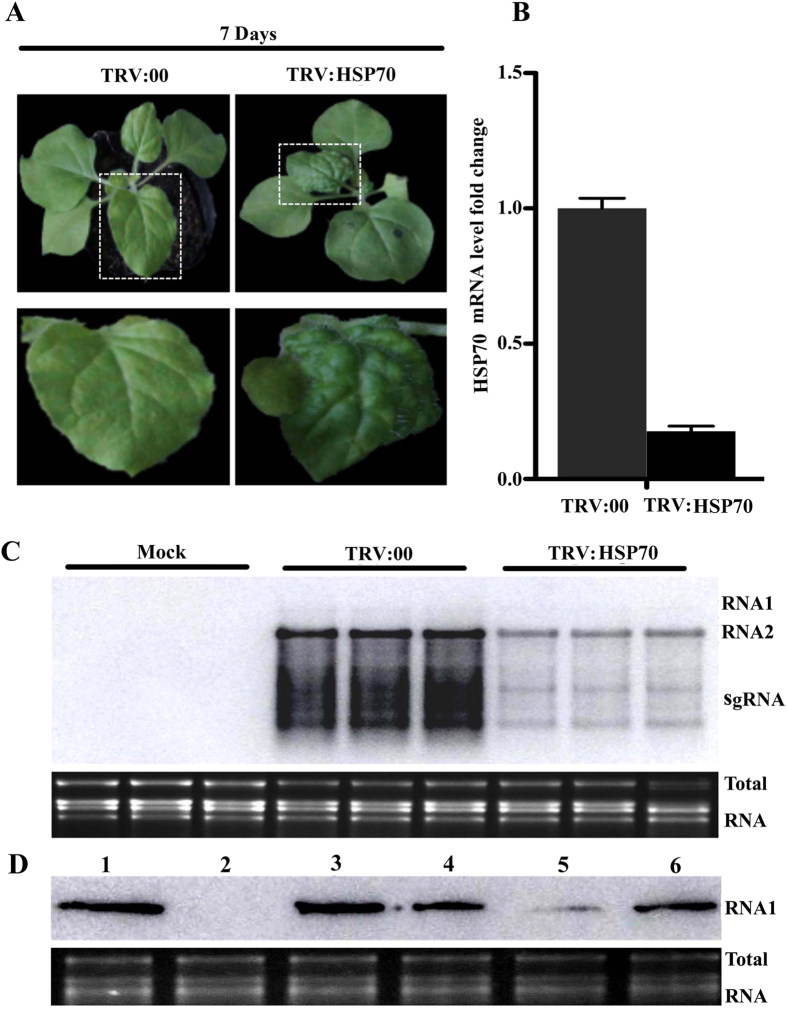
Expression levels of NbHSP70 are associated with the accumulation of CWMV RNAs in *N.benthamiana* plants. (**A**) Symptoms in *N. benthamiana* plants infiltrated with the tobacco rattle virus (TRV) vector harboring a partial fragment of *N. benthamiana* HSP70 (TRV:NbHSP70). The empty vector (TRV:00) was infiltrated and used as a control. Pictures were taken at 7 dpi. (**B**) qPCR analysis showing the expression levels of NbHSP70 in the *N. benthamiana* infiltrated with TRV:NbHSP70 compared with the control (TRV:00). Experiments were repeated three times. Bars represent the standard errors of the means. (**C**) Accumulation of CWMV RNAs analyzed by Northern blotting. Total RNAs were extracted from the inoculated leaves at 4 dpi. Ethidium bromide (EtBr)-stained rRNAs are shown as loading controls. (**D**) Accumulation of CWMV RNA1 analyzed by Northern blotting using an RNA1-specific probe. Lane 1, the total RNA extracted from a plant inoculated with CWMV RNA 1 and RNA 2. Lane 2, the total RNA extracted from the mock inoculated *N. benthamiana*. Lane 3, the total RNA extracted from the silencing plants co-inoculated with CWMV RNA1 and 35S:NbHSP70. Lane 4, the total RNA extracted from the control plants inoculated with TRV:00 and CWMV RNA1. Lane 5, the total RNA extracted from the NbHSP70 silenced plants inoculated with CWMV RNA1. Lane 6, the total RNA extracted from the plants inoculated with CWMV RNA1 only. Total RNAs were extracted from the inoculated leaves 4 dpi after inoculation. Ethidium bromide (EtBr)-stained rRNAs are shown as loading controls.

To further assess the potential function of HSP70 in CWMV infection, tobacco rattle virus (TRV)-induced gene silencing (VIGS) was used to investigate the effect of NbHSP70 silencing on CWMV infection in *N. benthamiana*. TRV vectors harboring a partial fragment of the NbHSP70 gene (TRV:NbHSP70) were constructed and agro-infiltrated into plants while the empty TRV vector (TRV:00) was used as control. The specific reduction of NbHSP70 transcripts was validated by qRT-PCR assays in plants infiltrated with TRV:HSP70 at 7 dpi and the distinct crinkles and abnormal veins were observed on the upper leaves of plants infiltrated with TRV:HSP70 at 10 dpi (Fig. 4A,B). Death of some young leaves and shoot apical meristems occurred at 14 dpi. The newly developed leaves were inoculated at 10 dpi with CWMV infectious clones by *Agrobacterium* infiltration and monitored for virus accumulation. Total RNA was extracted from the systemic leaves 4 days after CWMV inoculation (i.e. at 14 dpi). Northern blotting assays showed that the accumulation of CWMV RNAs was significantly reduced in the TRV:NbHSP70-infiltrated plants when compared with TRV:00 plants (Fig. 4C).

### Effect of NbHSP70 and TaHSP70 on accumulation of CWMV RNA1 in plants

CWMV has two genomic RNAs but only RNA1 encodes proteins required for viral replication^23^. To validate the effect of NbHSP70 on CWMV replication, full-length RNA1 clones were agro-inoculated onto the newly developed leaves of *N. benthamiana* in which the silencing phenotype had been fully established by prior inoculation with the TRV:NbHSP70 construct. *N. benthamiana* plants agri-inoculated with the empty TRV vector and the CWMV RNA1 clone were used as controls. Total RNA was extracted from the inoculated leaves at 4 dpi and used for Northern blotting assays with a RNA1-specific probe. Accumulation of RNA1 was reduced in NbHSP70-silenced plants (Fig. 4D, lane 5) when compared with that of non-silenced plants (Fig. 4D, lane 4). However, the accumulation of CWMV RNA1 was increased sharply in NbHSP70-silenced plants co-infiltrated with CWMV RNA1 and 35S:NbHSP70 (Fig. 4D, lane 3) or 35S:TaHSP70 constructs (data not shown), to a greater extent even than in plants inoculated only with the CWMV RNA1 clone (Fig. 4D, lane 6). Thus, silencing of NbHSP70 appeared to inhibit the accumulation of RNA1 but addition of NbHSP70 or TaHSP70 to the silenced plants enhanced RNA accumulation. Since there were similar adverse and advantageous effects of NbHSP70 on RNA1 alone and on the fully infective clones of CWMV, it appears that the function(s) of NbHSP70 and TaHSP70 could be closely related to the proteins encoded on CWMV RNA1.

### Distribution patterns of both NbHSP70 and TaHSP70 were affected by CWMV infection and by the RNA1 clone

**Figure 5:**
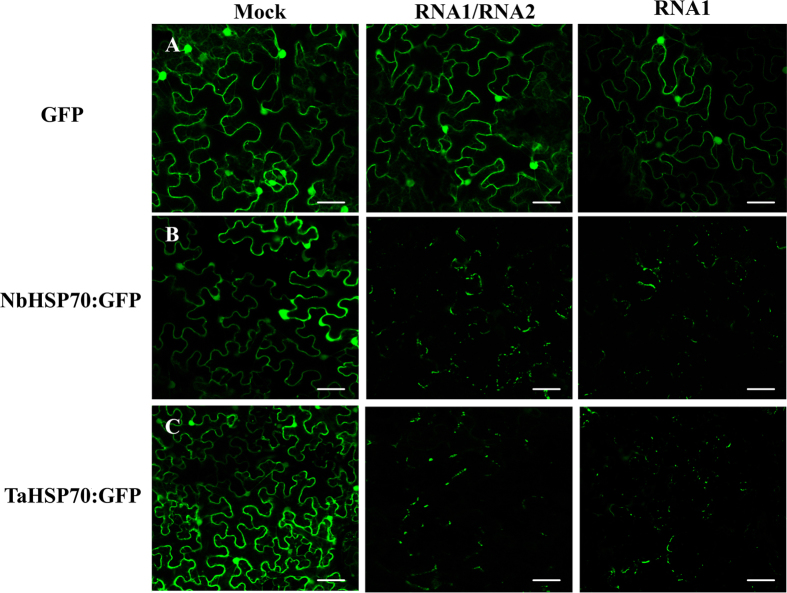
Localization of TaHSP70 or NbHSP70 was affected by CWMV infection. GFP fluorescence in healthy (Mock) or CWMV-infected *N. benthamiana* leaf epidermal cells agroinfiltrated with pCV-GFP-N1 (**A**), pCV-TaHSP70-GFP (**B**) and pCV-NbHSP70-GFP (**C**), respectively. The results were observed 72 h after infiltration. Scale bar, 50 μm.

To examine the sub-cellular localization of NbHSP70 and TaHSP70 proteins, constructs expressing NbHSP70 or TaHSP70 fused with eGFP at their C terminus (NbHSP70:GFP and TaHSP70:GFP) were constructed and introduced into *N. benthamiana* epidermal cells by *Agrobacterium* infiltration. At 3 dpi, GFP fluorescence was detected by confocal microscopy and found to be mainly in localized parts of the cytoplasm and nucleus of cells expressing NbHSP70:GFP or TaHSP70:GFP (Fig. 5B,C). In the control, the non-fused GFP was distributed generally in the cytoplasm and nucleus (Fig. 5A), consistent with previous reports^24^. However, in CWMV-infected cells, fluorescence of both GFP-fused proteins was limited to numerous granules of various sizes in the cytoplasm and no fluorescence was observed in the nucleus (Fig. 5), suggesting that the distribution pattern of both NbHSP70:GFP and TaHSP70:GFP was severely affected by CWMV infection.

Furthermore, when NbHSP70:GFP or TaHSP70:GFP were agro-infiltrated together with the CWMV RNA1 clone into *N. benthamiana* epidermal cells, a large number of irregular fluorescent granules were also observed in the cytoplasm (Fig. 5). By contrast, in cells of CWMV-infected plants either expressing the non-fused GFP or co-expressing NbHSP70:GFP or TaHSP70-GFP with the CWMV RNA2 clone, the fluorescence patterns were similar to those of the cells expressing non-fused GFP, NbHSP70:GFP, or TaHSP70-GFP alone (data not shown). These results suggest that CWMV infection modifies the subcellular distribution pattern of TaHSP70 and NbHSP70 and that the proteins encoded by CWMV RNA1 are responsible for this subcellular modification.

### The CWMV replicase interacts with NbHSP70 and TaHSP70 and the region aa 167–333 of the replicase is responsible for this interaction *in vivo* and *in vitro*

**Figure 6:**
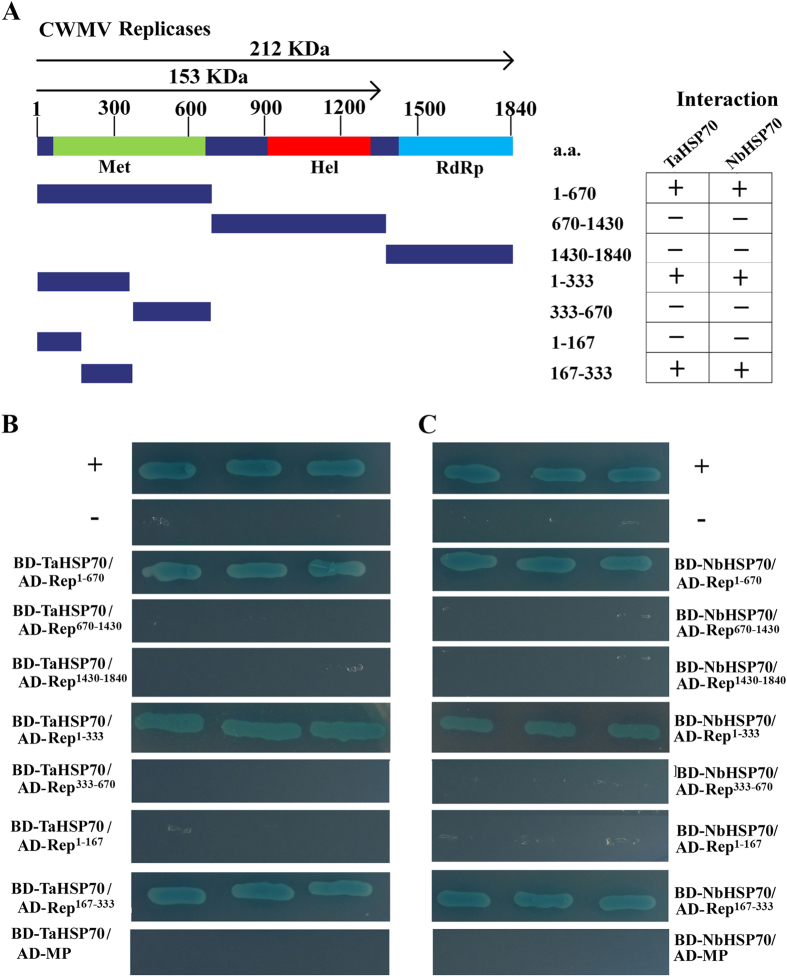
Interactions of TaHSP70 or NbHSP70 with CWMV replicase or MP in the yeast two hybrid system. (**A**) Seven truncated mutants, covering the three conserved domains (Met, Hel and RdRp) of the CWMV replicase, were designed for assays. The numbers denote CWMV replicase amino acid positions. The ability of CWMV replicase fragments to interact with TaHSP70 or NbHSP70 in YTH assays is shown on the right (+, positive; −, negative). (**B**) Yeast colonies expressing BD-TaHSP70 with AD-Rep^1–670^ and AD-Rep^167–333^ grew well on the selective medium, but those expressing AD-Rep^670–1430^, AD-Rep^1430–1840^, AD-Rep^1–167^, AD-Rep^333–670^ or AD-MP with BD-TaHSP70 did not. (**C**) Yeast colonies expressing BD-NbHSP70 with AD-Rep^1–670^ and AD-Rep^167–333^ grew well on the selective medium, but those expressing AD-Rep^670–1430^, AD-Rep^1430–1840^, AD-Rep^1–167^, AD-Rep^333–670^ or AD-MP with BD-NbHSP70d did not. Yeast co-transformed with BD-53 and AD-T, and BD-Lam and AD-T were used as the positive and negative controls, respectively.

To examine the interactions of NbHSP70 and TaHSP70 with the proteins encoded on CWMV RNA1, a series of yeast two-hybrid (YTH) assays were performed. On the basis of their predicted functional domains^17^, the replicase proteins were divided into three fragments, namely Rep^1–670^ (encoding aa 1–670, containing a domain for methyltransferase activity), Rep^670–1430^ (encoding aa 670–1430, containing a domain for helicase activity), and Rep^1430–1840^ (encoding aa 1430–1840, containing a domain for RNA-dependent RNA polymerase activity) (Fig. 6A). These three fragments and the full-length encoding region of the MP were then separately cloned into to the GAL4 activation domain (AD) vector pGADT7 as prey proteins AD-Rep^1–670^, AD-Rep^670–1430^, AD-Rep^1430–1840^, and AD-MP while both full-length coding regions of NbHSP70 and TaHSP70 were respectively cloned into the GAL4 DNA binding domain (BD) vector pGBKT7 as bait proteins BD-NbHSP70 and -TaHSP70. Expression of these fusion genes was under the control of galactose-inducible promoters. Combinations of plasmids expressing bait and prey proteins were then co-transformed into *S. cerevisiae* using the plasmid combinations BD-Lam/AD-T, AD/BD-NbHSP70 or AD/BD-TaHSP70 as negative controls and BD-53/AD-T as a positive control. The transformants expressing AD-Rep^1–670^/BD-NbHSP70 and AD-Rep^1–670^/BD-TaHSP70 grew well on the selective medium and turned as blue as the positive control (Fig. 6B,C). In contrast, no growth was observed in the transformants with any combination using prey plasmids AD-Rep^670–1430^, AD-Rep^1430–1840^ or AD-MP with bait plasmids BD-NbHSP70 or BD-TaHSP70, or in the negative controls (Fig. 6B,C). These results consistently indicated a strong interaction between the NbHSP70 or TaHSP70 proteins and the N-terminal region (aa 1–670) of the CWMV replicase. To determine the key domain for interaction, the terminal region (aa 1–670) of CWMV replicase was further divided into two smaller fragments, Rep^1–333^ and Rep^333–670^ for similar yeast two hybrid assays which showed that the crucial domain for interaction with HSP70 was within the N-terminal region (residues aa 1–333) of the replicase. This region was then divided again into two smaller fragments, Rep1–167 and Rep167–333, and the region aa 167–333 identified as crucial for its interaction with NbHSP70 or TaHSP70 (Fig. 6).

**Figure 7:**
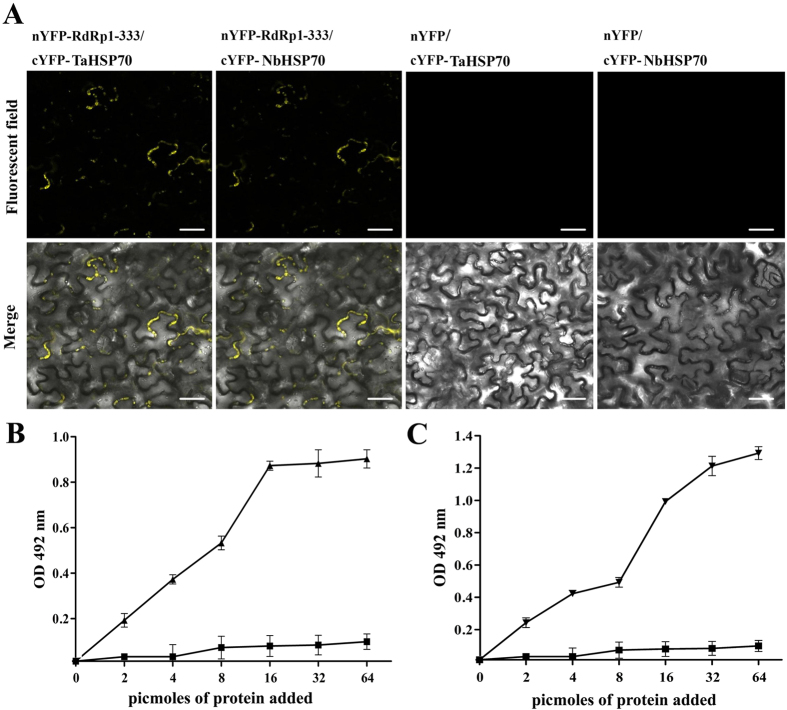
Interaction between the N-terminus of CWMV replicase and TaHSP70 or NbHSP70 *in vivo* and *vitro*. (**A**) Visualization of the interaction between Rep^1–333^ and TaHSP70 or NbHSP70 in *N. benthamiana* epidermal cells by BiFC assay. *N. benthamiana* leaves were co-infiltrated with recombinant BiFC vectors containing the constructs indicated above the images. The results were observed 48 h after infiltration. Scale bar, 50 μm. The fluorescent and merged images are depicted in the upper and lower panels, respectively. (**B**,**C**) Interaction of CWMV Rep^1–333^ with TaHSP70 and NbHSP70 proteins in ELISA-based binding assays. Wells of a microtiter plate were coated with 25 pmol of *E. coli* purified GST-tagged Rep^1–333^ protein and incubated with increasing amounts of *E. coli* purified 6×-histidine-tagged TaHSP70 protein (▲) or 6×-histidine-tagged NbHSP70 protein (▼), respectively. Retention of the complex was detected with polyclonal anti-His antibodies. The recombinant 6×-histidine tag was used alone as a control (■). Experiments were repeated three times. Bars represent the standard errors of the means.

To verify the YTH results, we then used bimolecular fluorescence complementation (BiFC) assays to test the *in vivo* interactions between the N-terminus (residues 1–333) of CWMV replicase and NbHSP70 or TaHSP70 in living plant cells. The coding sequence of Rep^1–333^ was cloned into the vector pCV-nYFP-C and that of NbHSP70 or TaHSP70 into pCV-cYFP-C to generate plasmids pCV-nYFP-Rep^1–333^, pCV-cYFP-NbHSP70 and pCV-cYFP-TaHSP70, respectively. Two pairs of combinations, pCV-nYFP-Rep^1–333^/pCV-cYFP-NbHSP70 and pCV-nYFP-Rep^1–333^/pCV-cYFP-TaHSP70, were generated, while the pairs of pCV-nYFP-Rep^1–333^/pCV-cYFP, pCV-nYFP/pCV-cYFP-TaHSP70 and pCV-nYFP/pCV-cYFP-NbHSP70 were used as the negative controls. These combinations were used to agro-infiltrate *N. benthamiana* leaves. Three days after agroinfiltration, the inoculated leaves were examined for YFP fluorescence using laser confocal scanning microscopy. As shown in Fig. 7A, strong reconstitution of YFP fluorescence was detected in leaf epidermal cells co-infiltrated with pCV-nYFP-Rep^1–333^/pCV-cYFP-NbHSP70 and pCV-nYFP-Rep^1–333^/pCV-cYFP-TaHSP70, whereas no fluorescence was observed in the negative controls.

To test for the direct interaction of NbHSP70 or TaHSP70 and the N-terminus of the replicase *in vitro*, an enzyme linked immunosorbent assay (ELISA)-based binding assay was then performed. For this purpose, Rep^1–670^ was bacterially expressed as a GST-tagged fusion GST-Rep^1–670^ (Figure S3) and NbHSP70 or TaHSP70 was expressed as a 6 × histidine-tagged fusion His-NbHSP70 and His-TaHSP70, respectively (Figure S3). ELISA plate wells were coated with purified GST-Rep^1–670^. The coated wells were then incubated with increasing concentrations of purified 6 × His-tagged NbHSP70 or TaHSP70. Complex retention was detected using an anti-His antiserum. Saturation binding curves were established for NbHSP70 (Fig. 7B) and TaHSP70d (Fig. 7C). No signal was detected when His-NbHSP70 or His-TaHSP70 was replaced with the His protein. These results confirmed that the replicase directly interacted with both NbHSP70 and TaHSP70 and the mechanisms of their interaction were similar.

### The viral replicase recruits NbHSP70 or TaHSP70 to the membrane within plant cells

**Figure 8:**
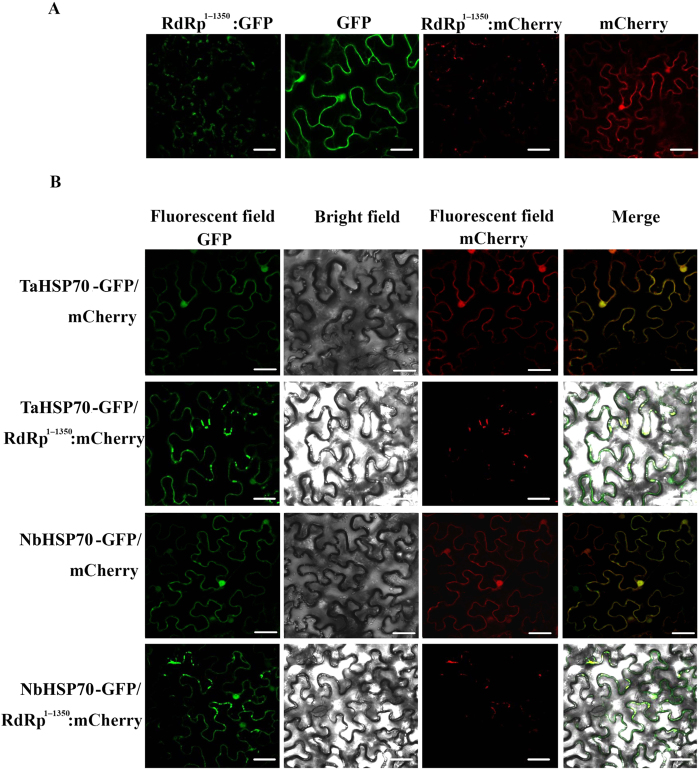
Localization of TaHSP70 or NbHSP70 was affected by expression of the N-terminus of CWMV Rep^1–1350^. (**A**) Sub-cellular localization of CWMV Rep^1–1350^ fused with GFP or mCherry in *N. benthamiana* leaf epidermal cells. (**B**) Epidermal cells of *N. benthamiana* transiently co-expressing Rep^1–1350^-mCherry and TaHSP70-GFP or NbHSP70-GFP. The results were observed 72 h after infiltration. Scale bar, 25 μm.

We next examined the subcellular distribution of the CWMV replicase when expressed alone or together with NbHSP70 or TaHSP70. The replicase (aa 1–1350, without the read through domain) was fused to the C terminus of green fluorescent protein (Rep^1–1350^-GFP) or of red fluorescent protein (Rep^1–1350^-mCherry) and introduced into *N. benthamiana* epidermal cells by *Agrobacterium* infiltration. When the fluorescence was observed by confocal microscopy, Rep^1–1350^-GFP (Fig. 8A) or Rep^1–1350^-mCherry (Fig. 8A) were distributed in the cytoplasm forming numerous granular aggregations or inclusion-like structures, which were different from the subcellular distribution of NbHSP70 and TaHSP70 when expressed alone (Fig. 5 Mock). When Rep^1–1350^-mCherry was co-expressed with NbHSP70:GFP or TaHSP70:GFP, Rep:mCherry appeared to be co-localized with NbHSP70:GFP or TaHSP70:GFP in most of the granular aggregations or inclusion-like structures within the co-transfected cells (Fig. 8B), a pattern similar to the subcellular distribution of NbHSP70 or TaHSP70 in the cells inoculated with RNA1 or RNA1/RNA2 of CWMV (Fig. 5 RNA1 and RNA1/RNA2) and shown in BiFC assays (Fig. 7A) but different from the subcellular distribution of NbHSP70 and TaHSP70 when expressed alone (Fig. 5 Mock) or co-expressed with mCherry in cells (Fig. 8), suggesting that the interaction is responsible for the differences in subcellular localization of NbHSP70 and TaHSP70. We also noted that the granular aggregations or inclusion-like structures formed in co-transinfected cells were similar in size and appearance to those of cells expressing Rep:mCherry alone. Based on these results, we formed a hypothesis that the viral replicase might recruit NbHSP70 or TaHSP70 into the granular aggregations or inclusion-like structures.

**Figure 9:**
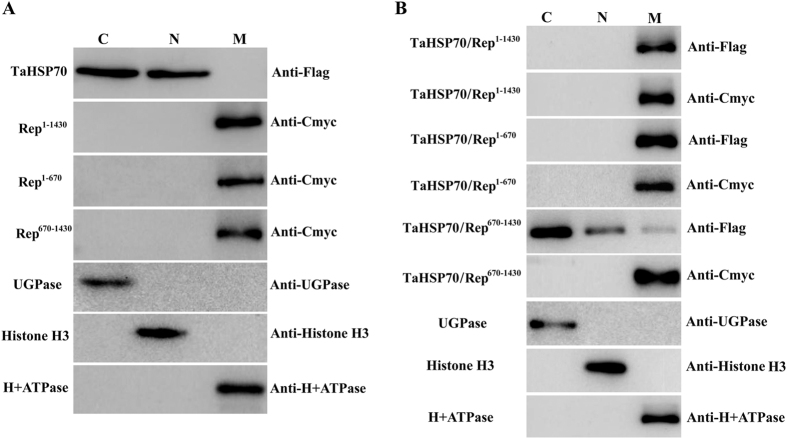
Subcellular fractionation assays of TaHSP70 and CWMV replicase or its truncated mutants expressed alone (**A**) or together (**B**) in *N. benthamiana* leaf epidermal cells. C, cytoplasm fraction; N, nuclear fraction; M, membrane fraction.

In all plant RNA viruses, the viral replicases are major components of the viral inclusions, which are thought to be related to the intracellular membrane system and to serve as a factory for viral replication or assembly^25^. To validate the hypothesis, we expressed TaHSP70 and the viral replicase or its truncated mutants alone or co-expressed a mixture of them and then performed subcellular fractionation assays as described previously. As shown in Fig. 9A, TaHSP70 was always detectable in both nuclear and cytoplasmic fractions when expressed alone; interestingly, Rep^1–1430^ and its truncated mutants, Rep^1–670^ and Rep^670–1430^, were detectable in membrane fractions but not in nuclear or cytoplasmic fractions when expressed alone, suggesting that the viral replicase is a membrane protein. However, TaHSP70 was detectable in the membrane fraction but not in nuclear or cytoplasmic fractions when co-expressed with Rep^1–1430^ and its truncated mutant Rep^1–670^. When co-expressed with Rep^670–1430^, TaHSP70 was detectable in all fractions (Fig. 9B). In another series of subcellular fractionation assays, NbHSP70 appeared to have a similar behavior to that of TaHSP70 (Data not shown). Taken together, these results supported the hypothesis that the viral replicase recruits NbHSP70 or TaHSP70 to the membrane fraction by specific and direct interaction and suggests that the interaction may be involved in the formation of functional inclusion-like structures.

## Discussion

HSPs are essential for cell viability, the cell cycle, apoptosis and the innate immunity pathway by preventing protein misfolding and aggregation, promoting protein complex assembly and disassembly, facilitating protein trafficking and activating the regulation of proteins in signal transduction pathways^2^,^3^,^30^. A HSP70 from wheat germ lysate has the ability to interact functionally with chaperone proteins of the animal kingdom to cooperate in protein folding^26^, suggesting that the HSP70 family has a highly-conserved function. Here TaHSP70 and NbHSP70 were shown to be expressed at relatively low levels under normal conditions, suggesting that this type of HSP70 could have functions in growth and development. The TRV-based silencing of NbHSP70 resulted in distinct crinkles and abnormal veins on leaves of *N. benthamiana* plants at 10 dpi (Fig. 3A,B) and even the death of young leaves and shoot apical meristem at 14 dpi, which were effects similar to those seen in *N. benthamiana* plants with PVX-based silencing of NbHSP70c-1 (accession number: AB105430; partial sequence: 1203 nt; 383 aa), a cytoplasmic member of the HSP70 family^27^,^28^. Sequence analysis showed that these two HSP70s had 86.6% nucleotide and 79.6% amino acid identity and their similar silencing phenotypes suggested that they might be required for normal growth and development of *N. benthamiana* and that they could have redundant functions. NbHSP70c-1 has been shown to be involved in plant defense signaling pathways and its silencing reduces the expression of defense genes, thus compromising plant resistance to bacterial or fungal disease^27^. Silencing of NbHSP70c-1 impaired viral multiplication and movement of tobacco mosaic virus (TMV), reflecting the distinct roles of plant HSP70s in viral, as opposed to bacterial or fungal infection^28^. In this study, both VIGS silencing and treatment with quercetin, a specific inhibitor of HSP70^20^, significantly decreased the accumulation of CWMV genomic RNAs. In these and overexpression assays, we also noted that the expression level of the HSP70s appeared to be positively correlated with the accumulation of CWMV genomic RNA in *N. benthamiana.* This supports the idea that the HSP70s are functionally redundant during viral infection although there may be some differences in function since, for example, NbHSP70c-1 was thought to be a cytoplasmic protein whereas in our experiments NbHSP70 and TaHSP70 were present in both the cytoplasm and nucleus.

Infection and replication of all viruses greatly depends on host proteins, And HSP70s are one of the best studied of such host factors^1^,^29^,^30^. Members of the HSP70 family can participate in various steps of the viral infection cycles, including viral RNA replication, viral protein folding, virion assembly, and viral movement, perhaps depending on the different virus-host systems^1^,^31^,^32^. In geminiviruses, HSP70s can interact with the coat protein (CP)^33^ or movement protein (MP)^34^; in potexviruses, HSP70 has also been implicated in an interaction with CP^35^. However, we found no interaction between NbHSP70 or TaHSP70 and the MP or CP of CWMV, suggesting that these HSP70s may not be directly involved in CWMV movement or other specific functions of the MP and CP. Previous studies have shown that HSP70s interact with different viral replicases and are involved in replication and transcription of some eukaryotic RNA viruses^36^,^37^,^38^,^39^,^40^,^41^. During potyviral infection, HSP70 interacts with the viral replicase and regulates gene expression in co-operation with CPIP^31^,^38^,^42^. In our experimental system, TaHSP70 or NbHSP70 was required for accumulation of CWMV genomic RNAs. This is consistent with previous studies showing the importance of HSP70s for efficient viral infection^38^,^43^,^44^ and which may indicate that HSP70s regulate replication or transcription. To our knowledge, this is the first host factor identified to play a crucial role in furoviral infection.

In host cells, viruses often induce host membrane rearrangements to form novel cytoplasmic vesicular compartments where viral RNA, replicases, or replicase-associated proteins interact with host factors and assemble the viral replicase complexes (VRC) for their own efficient replication^45^. VRC studies have provided important information about viral pathogenicity, virus-host interactions and the mechanisms regulating viral genome replication^45^,^46^. In tombusviruses, HSP70 promotes the subcellular transport of replicase proteins to intracellular membranes and is required for the *in vitro* activity or assembly of the viral replicase complex (VRC)^40^,^47^. HSP70s have also been found to be necessary for the proper conformational arrangement of the replicase and its assembly at sites of viral replication^48^,^49^,^50^,^51^. In this study, the viral replicase, a major component of VRC, could form granular aggregations or viral inclusion-like structures in membranes when expressed alone, suggesting that it may be the skeleton or organizer for VRC formation. NbHSP70 and TaHSP70 appeared to be translocated into the intracellular membrane when co-expressed with the CWMV replicase. In addition, NbHSP70 or TaHSP70 also formed granules or viral inclusion-like structures in CWMV-infected cells. All of these results suggests that the host factor is a component of the VRC and that it is recruited by the replicase during CWMV infection. The regulated and re-localized molecular chaperones may regulate the conformation or replication activity rather than formation of VRC.

## Materials and Methods

### Plants, virus inoculation and RNA extraction

CWMV-infected wheat plants with typical mosaic symptoms were collected from a disease nursery in Yantai city, Shangdong province, China. *Nicotiana benthamiana* plants at the six-leaf stage were mechanically inoculated with mixtures of *in vitro* transcripts from RNA1 and RNA2, as described previously^19^. Systemic infection was confirmed by RT-PCR 14 days after inoculation. All *N. benthamiana* plants were grown in a cabinet at 17 °C, with 16 h light/8 h dark and 70% r.h. Leaves were collected from the infected plants, frozen and stored at −80 °C until use. Total RNAs were extracted from plants using the Trizol Reagent (Invitrogen) and stored at −80 °C.

### Western blot analysis

About 0.1 g leaf tissue frozen in liquid nitrogen was ground to a fine powder and thawed in plant protein extraction buffer (Sigma) containing Protease Inhibitor Cocktail Tablets (Roche; 1 Tablet/50 ml). The mixture was centrifuged at 18,000 g for 10 min at 4 °C. 20 μg of each protein sample was boiled in SDS-PAGE buffer at 100 °C for 10 min and centrifuged at 18,000 g for 5 min. The supernatant was separated in 15% SDS-PAGE, followed by Western blot analysis using rabbit polyclonal antibody against purified CWMV particles or other commercial antibodies as the primary antibodies (1:5000) and anti-rabbit goat IgG conjugated with alkaline phosphatase as the secondary antibody (1:10000) following the method described previously^19^.

### Virus-induced gene silencing (VIGS)

Tobacco Rattle Virus (TRV)-based vectors, pYL196 (RNA1) and pTRV (RNA2), were kindly provided by Dr Yule Liu, Tsinghua University, Beijing, China^52^. The partial sequence of NbHSP70 was amplified by RT-PCR with the primer pair Nb70F/Nb70R (Table S1) from a leaf cDNA library of *N. benthamiana* plants, ligated into pTRV vector digested with XbaI and KpnI restriction enzymes, producing vector TRV:NbHSP70 for silencing the expression of endogenous NbHSP70 in *N. benthamiana* plants. The empty vector, TRV:00, was used for the control treatment. Agro-infiltration for VIGS was performed as previous described^53^. The upper leaves were inoculated with CWMV at 7–10 dpi when silencing was fully established.

### Northern blotting assays

Northern blot analysis was performed essentially as previously described^19^. Total RNAs were extracted from leaf tissues using TRIzol reagent (Invitrogen) and treated with DNase (TAKARA). 3 μg of total RNAs were separated on a denaturing 2% formaldehyde agarose gel and transferred to Hybond-N+ membranes (Amersham Bioscience) using 20 × SSC. The RNAs were cross-linked to the membrane matrix by UV for 45 s. Northern blotting for assays of CWMV genomic RNAs were carried out using DIG High Prime DNA Labeling and Detection Starter Kit II (Roche). DNA oligonucleotides complementary to the 3′-terminus of the CWMV genome or specifically complementary to CWMV RNA1 were labeled with digoxigenin (DIG) at their 3′ ends using the DIG Oligonucleotide Tailing Kit (Roche) and then purified using a G25 Sephadex column (GE). Membranes were prehybridized for 2 h and hybridized overnight at 42 °C using the DIG Luminescent Detection Starter Kit for Nucleic Acids (Roche). The hybridization signals were visualized by the Amersham Imager 600 (GE). All these procedures followed the manufacturers’ instructions.

### Yeast two-hybrid (YTH) assays

The yeast GAL4 binding domain vector pGBKT7 and GAL4 activation domain vector pGADT7 (Clontech, Palo Alto, CA) were used for yeast two hybrid (YTH) assays. To construct plasmids for YTH analysis, the coding sequence of TaHSP70 (GenBank accession no. AF005993) and NbHSP70 (GenBank accession no. KX912913) were amplified separately by PCR from *Triticum aestivum* and *N. benthamiana* cDNA libraries using primer pairs BD-Ta70N/BD-Ta70C and BD-Nb70N/ BD-Nb70C (Table S1). The amplified fragments were digested with NdeI/BamHI and cloned into similarly digested yeast GAL4 binding domain pGBKT7 vectors creating the recombinant bait plasmids BD-TaHSP70 and BD-NbHSP7d, respectively. In further YTH assays, a series of truncated mutants of the CWMV replicase (aa 1–670, 670–1430, 1430–1840, 1–333, 333–670, 1–167, and 167–333) were amplified separately from CWMV cDNA using the primer pairs shown in Table S1. These amplified fragments were digested with XmaI /BamHI and then inserted into pGADT7 vector digested with XmaI/BamHI creating the recombinant prey plasmids, AD-Rep^1–670^, -Rep^670–1430^, -Rep^1430–1858^, -Rep^1–333^, -Rep^333–670^, -Rep^1–167^, and -Rep^167–333^, respectively.

Yeast transformation was conducted in accordance with the recommended procedures (Matchmaker Gold Yeast Two-Hybrid System; Yeastmaker Yeast Transformation System 2, Clontech). Briefly, bait and prey plasmids were co-transformed into *S. cerevisiae* strain Y2HGold. The co-transformants were first plated on SD/-Ade/-His/-Leu/-Trp medium, and yeast colonies that grew on the auxotrophic medium were then tested for α-galactosidase activity on SD/-Ade/-His/-Leu/-Trp/X-α-Gal/ AbA medium. BD-53 and AD-T were also co-transformed as a positive control, while BD-Lam and AD-T were co-transformed as a negative control. Three independent experiments were performed to confirm the results.

### Expression and purification of recombinant proteins in *E. coli*

To construct the vector coding for the histidine-tagged full-length NbHSP70 and TaHSP70 fusion proteins, NbHSP70 and TaHSP70 were amplified by PCR from cDNA libraries prepared from leaves of *N. benthamiana* or *Triticum aestivum* using primers pET-Nb70N/pET-Nb70C and pET-Ta70N/pET-Ta70C (Table S1), respectively. The amplified fragments were digested with BamHI/NotI and cloned into digested pET28a vector (Novagen). For construction of the vector coding for the C-terminus GST fusion of Rep^1–670^ fusion protein, CWMV Rep^1–670^ sequences were amplified from a CWMV cDNA library using the primer pair p6P1Rep^1–670^ N / p6P1Rep^1–670^ C (Table S1). The amplified fragments were digested with BamHI/EcoRI and cloned into digested pGEX-6P1 vector (GE Healthcare).

For purification of GST-tagged Rep^1–670^, or the GST tag itself, a 20 ml overnight culture of *E. coli* BL21 cells containing the recombinant plasmid pGEX-Rep^1–670^ or pGEX6P1 vector alone was used to inoculate 100 ml of LB media containing 75 μg/ml of carbenicillin. Cells were grown at 37 °C to an OD_600_ of 0.4. Protein expression was induced with 0.1 mM of IPTG for 3 h for Rep^1–670^-GST and GST. Bacterial cells were re-suspended in 20 ml of phosphate buffered saline solution (PBS; 4.3 mM Na_2_HPO_4_, 1.47 mM KH_2_PO_4_, 137 mM NaCl, 2.7 mM KCl, pH 7.3) supplemented with 1 mM DTT, 40 mg of lysozyme, and 1× complete EDTA-free protease inhibitor. The cells were disrupted by sonication and supplemented with 1% Triton-X-100. Lysate was centrifuged at 30,000 × g for 30 min at 4 °C. The supernatant was filtered through a 0.45 μm Millex HV PVDF filter (Millipore) and used for affinity purification of either Rep^1–670^-GST or GST on glutathione sepharose 4B (GE Healthcare) according to the manufacturer’s protocol. For purification of histidine-tagged TaHSP70 and NbHSP70, a 5 ml overnight culture of *E. coli* BL21(DE3) cells containing the recombinant plasmid pET28(a)-TaHSP70 or pET28(a)-NbHSP70 was used to inoculate 100 ml of LB media containing 50 μg/ml of kanamycin. Cells were grown at 37 °C to an OD_600_ of 0.4 to 0.6. Protein expression was induced with 1 mM of IPTG for 2 h at 37 °C. Bacterial cells were re-suspended in 5 ml of buffer A (50 mM sodium phosphate buffer pH 8.0, 300 mM NaCl, 5 mM β-mercaptoethanol, 0.1% Tween-20, 10% glycerol, 1× complete EDTA-free protease inhibitor). The cells were disrupted by sonication and the resulting lysate centrifuged at 30,000 × g for 30 min at 4 °C. The supernatant was used for immobilized metal affinity purification on Talon resin (BD Biosciences). Resin was washed with buffer A supplemented with 10 mM imidazole and proteins eluted with buffer B (20 mM Tris-HCl pH 7.5, 300 mM NaCl, 250 mM imidazole, 2.5 mM DTT). Concentration of all recombinant proteins produced was measured using a Bradford assay (Bio-Rad) using bovine serum albumin as standard. Protein purity and molecular weight were assessed by Coomassie staining and immunoblot analysis using monoclonal anti-histidine, monoclonal anti-GST.

### ELISA-based binding assays

Replicase protein (100 μl of protein at 50 ng /μl in PBS buffer) was adsorbed to wells of a polystyrene plate (Costar) by incubation at 4 °C for 2 h and wells were blocked with 5% milk PBS solution for 2 h at room temperature. TaHSP70-His or NbHSP70-His proteins were diluted in PBS with 1% milk and 0.1% Tween-20 and incubated for 2 h at 4 °C in the coated wells. Detection of retained protein was achieved with a mouse monoclonal anti-His-tag antibody (Abcam, England) and horseradish peroxidase-coupled goat anti-mouse immunoglobulin (Abcam, England). Between each incubation, wells were washed three times with PBS supplemented with 0.05% Tween-20. Enzymatic reactions were performed in 100 μl of OPD citrate buffer (50 mM citric acid, 100 mM sodium phosphate dibasic, pH 5.0, 0.5 mg/ml o-phenylenediamine dihydrochloride, and 0.1% H_2_O_2_) and stopped with a solution of 3M H_2_SO_4_. Absorbance was measured at 492 nm. The S.E.M. was calculated for three biological replicates from a minimum of three technical replicates.

### Sub-cellular localization and BiFC assay

For subcellular localization analyses, a series of recombinant plasmids including Rep^1–1350^-GFP, Rep^1–1350^-mCherry, NbHSP70-GFP and TaHSP70-GFP were constructed by the Gateway technology according to the manufacturer’s instructions (Invitrogen). The first PCR used the primer pairs Rep^1–1350^-GFPN/Rep^1–1350^-GFPC, Rep^1–1350^–mCherryN/Rep^1–1350^–mCherryC, Nb70-GFPN/Nb70-GFPC and Ta70-GFPN/Ta70-GFPC (Table S1). The second PCR was performed with the primers attB1 and attB2 and the amplified product of the first PCR as the template. The amplified product was then introduced into pDONR207 by the BP reaction and the entry clones pENTR-Rep^1–1350^-GFP, pENTR-Rep^1–1350^-mCherry, pENTR-NbHSP70-GFP and pENTR-TaHSP70-GFP were constructed. Finally, the LR clonase reaction was used to transfer Rep^1–1350^, NbHSP70 and TaHSP70 fragments from the entry clones to the destination vector and recombinant plasmids including pMDC43-Rep^1–1350^, pGWB5C-Rep^1–1350^, pGWB5C-NbHSP70 and pGWB5C-TaHSP70 were constructed. These recombinanats were used to transform competent *E.coli* strain DH5α using heat shock and selected on a medium containing 50 μg/ml kanamycin and 50 μg/ml hygromycin.

The BiFC vectors pCV-nYFP-C and pCV-cYFP-C (for split YFP N- terminal/C-terminal fragment expression) were previously constructed in our laboratory. For BiFC assays, the coding sequence of the N-terminal fragment (residues 1–670) of CWMV replicase was amplified using primer pair BRepF/BRepR (Table S1) and cloned into pCV-nYFP-C as a fusion with the N-terminal fragment of YFP via the BamHI/SacI sites, forming pCV-nYFP-Rep^1–670^. The full-length coding sequences of TaHSP70 and NbHSP70 were amplified by PCR using primer pairs BTa70F/BTa70R and BNb70F/BNb70R (Table S1), respectively. PCR products were cloned into the BamHI/SacI or BamHI/NotI sites of pCV-cYFP-C as a fusion with the C-terminal fragment of YFP, resulting in pCV-cYFP-TaHSP70 and pCV-cYFP-NbHSP70.

The recombinant binary constructs were introduced into *Agrobacterium tumefaciens* strain C58C1 by electroporation (Bio-Rad Gene Pulser, 0.2 cm cuvettes, 25 microF, >2.5 kV). Agroinfiltration was done as described^24^. Briefly, cultures of C58C1 harbouring a relevant binary plasmid were grown in YEP medium containing rifampicin (50 μg/ml) and kanamycin (100 μg/ml) at 28 °C for 16 h. For the BiFC assay, C58C1 strains containing the BiFC plasmids with pCV-nYFP-Rep^1–670^/pCV-cYFP-TaHSP70 and pCV-nYFP-Rep^1–670^/pCV-cYFP-NbHSP70 were re-suspended and adjusted to an OD_600_ of 1.0:1.0 with infiltration medium (10 mM MES, pH 5.6, 10 mM MgCl_2_, 200 mM acetosyringone) before leaf infiltration, while the combinations pCV-nYFP-Rep^1–670^/pCV-cYFP, pCV-nYFP/pCV-cYFP-TaHSP70 and pCV-nYFP/pCV-cYFP-NbHSP70 were used as the negative controls. For sub-cellular localization, *Agrobacterium* cultures containing pGWB5C-Rep^1–1350^, pGWB5C-Rep^1–1350^, pGWB5C-NbHSP70 and pGWB5C-TaHSP70 were re-suspended and diluted to an OD_600_ of 0.6 before leaf infiltration. The cell suspensions were incubated at room temperature for 2 to 4 h and then used to infiltrate 5- to 6-week-old *N. benthamiana* leaves. Expression of fluorescent proteins was examined at 48 h post agroinfiltration^54^.

### Subcellular fractionation

Plant nuclear and cytoplasmic fractionation was performed using the Plant nuclei and cytoplasmic protein extraction kit (BestBio, Shanghai) according to the manufacturer’s protocol. Membrane fractionation assays were performed as previously described^55^. Briefly, plant material was ground to fine powder under liquid nitrogen. 2 × volumes of homogenization buffer (Sorbitol 0.5 M, EDTA 10 mM, PVP 40 0.5%, Protease inhibitor cocktail) were added and debris was removed by centrifugation at 8000 g for 15 min at 4 °C. After centrifugation at 100 000 g for 30 min at 4 °C, the supernatant corresponds to the soluble (S100) and the pellet to the particulate (P100) fraction. For the re-solubilization test, the P100 fraction was re-suspended in 5 × volume of the same buffer supplemented with detergent, incubated for 30 min on ice and centrifuged at 100 000 g for 30 min at 4 °C. The supernatant corresponds to the S100 and the pellet to the P100. The S100 fraction was precipitated before analysis. The proteins were pelleted at 5000 g for 5 min at 4 °C, washed with 80% acetone and finally re-suspended in 300 μl of resuspension solution (3% SDS; 62.5 mM TrisHCl pH 6.8; 10% glycerol). The samples were incubated at 65 °C for 10 min, centrifuged at 13 000 g for 5 min and the supernatant was kept for quantification and analysis. Ten micrograms were diluted in 1:5 SDS loading buffer and subjected to SDS-PAGE and immunoblotting.

### Confocal microscopy

Fluorescence analysis was performed using a Leica TCS SP5 confocal laser scanning microscope (Leica Microsystems, Heidelberg, Germany). GFP was excited at 488 nm and the emitted light captured between 500–550 nm, YFP was excited at 514 nm and the emitted light captured between 530–600 nm, and mCherry was excited at 561 nm and emission light captured between 570–630 nm. For analysis of co-localization assays, multi-tracking was used to prevent emission cross-talk between the channels. Images were captured digitally and handled using the Leica TCS software. Post-acquisition image processing was done with Adobe Photoshop version 7.0 software (Adobe Systems Inc., San Jose, CA, USA).

## Additional Information

**How to cite this article**: Yang, J. *et al*. A furoviral replicase recruits host HSP70 to membranes for viral RNA replication. *Sci. Rep.* **7**, 45590; doi: 10.1038/srep45590 (2017).

**Publisher's note:** Springer Nature remains neutral with regard to jurisdictional claims in published maps and institutional affiliations.

## Supplementary Material

Supplementary Tables and Figures
