# Supplementary material for: A furoviral replicase recruits host HSP70 to membranes for viral RNA replication
Source: Sci Rep. 2017 Apr 3;7:45590. doi: 10.1038/srep45590 (PMC5377427; doi:10.1038/srep45590)

# A furoviral replicase recruits host HSP70 to membranes for viral RNA replication

**Jian Yang [1], Fen Zhang [1, 2], Nian-Jun Cai [1, 2], Ne Wu [1, 3], Xuan Chen [1, 3], Jing Li [1], Xiang-Feng Meng [4], Tong-Quan Zhu [4], Jian-Ping Chen [1, *], Heng-Mu Zhang [1, 2, *]**

[1] State Key Laboratory Breeding Base for Zhejiang Sustain Pest and Disease Control; MOA and Zhejiang Key Laboratory of Plant Protection and Biotechnology, Institute of Virology and Biotechnology, Zhejiang Academy of Agricultural Sciences, Hangzhou 310021, China

[2]College of Chemistry and Life Science, Zhejiang Normal University, Jinhua 321004, China;

[3] Zhejiang Agriculture and Forest University, Linan 311300, China

[4] Zhumadian Academy of Agriculture Sciences, Zhumadian 463000, China

* Corresponding authors, E-mail: zhhengmu@tsinghua.org.cn; jpchen2001@126.com

**Running title:** A furoviral replicase recruits HSP70 to membranes for replication

**Supplementary materials**

**Supplementary Table**

**Table S1 Primers used in this study.**

**Supplementary Figures**

**Figure S1** Western blotting assays showing inhibition of NbHSP70 by quercetin treatment (A) and over-expression of TaHSP70 by Agrobacterium infiltration of 35S:TaHSP70 (B) in *N. benthamiana* plants.

**Figure S2** Sequence alignment of TaHSP70 and NbHSP70 showing that they are highly conserved.

**Figure S3** Recombinant expression of GST-tagged Rep$^{1\text{-}333}$ (GST-Rep$^{1\text{-}333}$) and 6 x His-tagged TaHSP70 (His-TaHSP70) in *E. coli* BL21DE3 for ELISA-base binding assays. M, protein Marker; Lane 1, Protein sample extracted from *E. coli* BL21DE3 expressing GST-Rep$^{1\text{-}333}$; Lane 2, Protein sample extracted from *E. coli* BL21DE3; Lane 3, Protein sample extracted from *E. coli* BL21DE3 expressing His-TaHSP70. White arrows point to the recombinant proteins GST-Rep$^{1\text{-}333}$ and His-TaHSP70, respectively.

# Table S1 Primers used in this study.

| Primers | Sequences (5'-3') | Restriction sites (underlined) | The purpose of the primers |
|---|---|---|---|
| Nb70F | CTAGTCTAGATATGCATACAACATGAGG | XbaI | Vector TRV:HSP70 |
| Nb70R | CGGGGTACCTTAGTCGACCTCCTCAAT | KpnI | |
| BDTa70N | GGAATTCCATATGATGGCCAAGGGCGAGGGG | NdeI | Bait plasmid BD-TaHSP70 |
| BDTa70C | CGCGGATCCGTCCACCTCC TCGATCTT | BamHI | |
| BDNb70N | GGAATTCCATATGATGGCAGGAAAAGGTGAA | NdeI | Bait plasmid BD-NbHSP70 |
| BDNb70C | CGCGGATCCGTCGACCTCC TCAATCTT | BamHI | |
| ADRep$^{1-670}$N | TCCCCACCCGGGATGCCGATCGATAGCAGT | XmaI | Prey plasmid AD- Rep$^{1-670}$ |
| ADRep$^{1-670}$C | CGCGGATCCTCCTTTCATTCACATGGAC | BamHI | |
| ADRep$^{670-1430}$N | TCCCCACCCGGGAGGAGAAATACTTCTGAT | XmaI | Prey plasmid ADRep$^{670-1430}$ |
| ADRep$^{670-1430}$C | CGCGGATCCCCAAACCCCGCTTGTCTTG | BamHI | |
| ADRep$^{1430-1858}$N | TCCCCACCCGGGTTGGTACCGGTGATGCGG | XmaI | Prey plasmid ADRep$^{1430-1858}$ |
| ADRep$^{1430-1858}$C | CGCGGATCCCCCATTCAAAGTTCCTATC | BamHI | |
| ADRep$^{1-167}$N | TCCCCACCCGGGATGCCGATCGATAGCAGT | XmaI | Prey plasmid ADRep$^{1-167}$ |
| ADRep$^{1-167}$C | CGCGGATCCTGGGTATCTCGTCCTTCTC | BamHI | |
| ADRep$^{167-333}$N | TCCCCACCCGGGCCCACCGAAGTCGGTGAA | XmaI | Prey plasmid ADRep$^{167-333}$ |
| ADRep$^{167-333}$C | CGCGGATCCCGTCCGTCATTGTGAAGAA | BamHI | |
| ADRep$^{333-670}$N | TCCCCACCCGGGGACGCAACGGAAGCGAGG | XmaI | Prey plasmid ADRep$^{333-670}$ |
| ADRep$^{333-670}$C | CGCGGATCCCTTCATTCACATGGACCAT | BamHI | |
| pETNb70N | CGCGGATCCATGGCAGGAAAAGGTGAA | BamHI | Plasmid pET28(a)-Nb70 |
| pETNb70C | ATTTGCGGCCGCAGTCGACCTCCTCAATCTT | NotI | |
| pETTa70N | CGCGGATCCATGGCCAAGGGCGAGGGG | BamHI | Plasmid pET28(a)-Ta70 |
| pETTa70C | ATTTGCGGCCGCAGTCCACCTC CTCGATCTT | NotI | |
| p6P1Rep$^{1-670}$ N | CGCGGATCCATGCCGATCGATAGCAGT | BamHI | Plasmid pGEX-Rep$^{1-670}$ |
| p6P1Rep$^{1-670}$ C | CCGGAATTCTTAATTTCTCCTTTCATTCAC | EcoRI | |
| Rep$^{1-1350}$-GFPN | AAAAAGCAGGCTCCATGCCGATCGATAGCAGT | | Plasmids Rep$^{1-1350}$-GFP |
| Rep$^{1-1350}$-GFPC | AGAAAGCTGGGTCTTTGTCGAATTCGGGACA | | |
| Rep$^{1-1350}$-mCherryN | AAAAAGCAGGCTCCATGCCGATCGATAGCAGT | | Plasmids Rep$^{1-1350}$-mCherry |
| Rep$^{1-1350}$-mCherryC | AGAAAGCTGGGTCTTTGTCGAATTCGGGACA | | |
| Nb70-GFPN | AA AAAGCAGGCTCCATGGCAGGAAAAGGT | | Plasmids NbHSP70-GFP |
| Nb70-GFPC | AGAAAGCTGGGTCGTCGACCTC CTCAATCTT | | |
| Ta70-GFPN | AA AAAGCAGGCTCCATGGCCAAGGGCGAG | | Plasmids TaHSP70-GFP |
| Ta70-GFPC | AGAAAGCTGGGTCGTCCACCTC CTCGATCTT | | |
| attB1 | ACAAGTTTGTACAAAAAAGCAGGCTCC | | Gateway vector |
| attB2 | ACCACTTTGTACAAGAAAGCTGGGTC | | |
| BRepF | CGCGGATCCATGCCGATCGATAGCAGT | BamHI | Plasmids pCV-nYFP-Rep$^{1-670}$ |
| BRepR | CGAGCTCCCTTTCATTCACATGGAC | SacI | |
| BTa70F | CGCGGATCCATGGCCAAGGGCGAGGGG | BamHI | Plasmids pCV-cYFP-TaHSP70 |
| BTa70R | CGAGCTCGTCCACCTC CTCGATCTT | SacI | |
| BNb70F | CGCGGATCCATGGCAGGAAAAGGTGAA | BamHI | Plasmids pCV-cYFP-NbHSP70 |
| BNb70R | ATTTGCGGCCGCGTCGACCTCCTCAATCTT | NotI | |

**Figure S1**

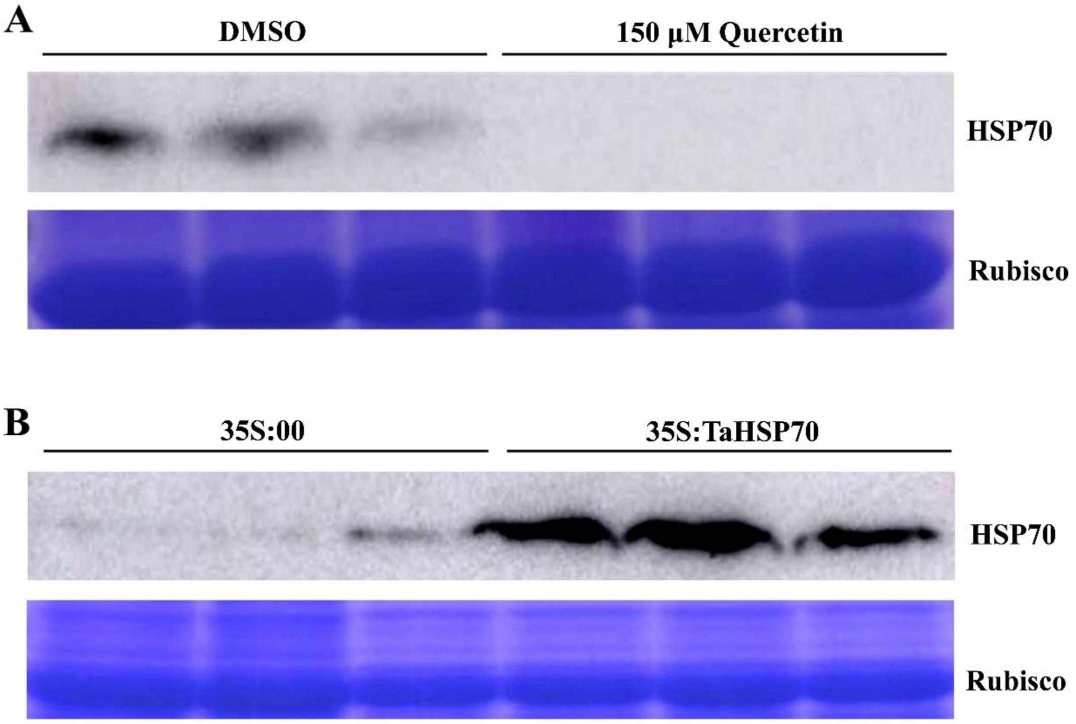
A
DMSO
150 µM Quercetin
HSP70
Rubisco
B
35S:00
35S:TaHSP70
HSP70
Rubisco

**Figure S2**

```
  NbHSP70 MAGKGEGPAIGIDLGTTYSCVGVWQHDRVEIIANDQGNRTTPSYVGFTDTERLIGDAAKNQVAMNPINTVFDAKRLIGRRFADASVQSDIKHWPFKVIPG 100
  TaHSP70 MA-KGEGPAIGIDLGTTYSCVGVWQHDRVEIIANDQGNRTTPSYVAFTDTERLIGDAAKNQVAMNPTNTVFDAKRLIGRRFSDPSVQSDMKLWPFKVIPG 99
Consensus MA KGEGPAIGIDLGTTYSCVGVWQHDRVEIIANDQGNRTTPSYV FTDTERLIGDAAKNQVAMNP NTVFDAKRLIGRRF D SVQSD K WPFKVIPG 93

  NbHSP70 PGDKPMIVVNYKGEEKQFAAEEISSMVLIKMREIAEAFLGSTVKNAVVTVPAYFNDSQRQATKDAGVIAGLNVLRIINEPTAAAIAYGLDKKATSVGEKN 200
  TaHSP70 PADKPMIVVNYKGEEKQFAAEEISSMVLIKMREIAEAFLGNSVKNAVVTVPAYFNDSQRQATKDAGAIAGLNVLRIINEPTAAAIAYGLDKKATSTGEKN 199
Consensus P DKPMIVVNYKGEEKQFAAEEISSMVLIKMREIAEAFLG  VKNAVVTVPAYFNDSQRQATKDAG IAGLNVLRIINEPTAAAIAYGLDKKATS GEKN 188

  NbHSP70 VLIFDLGGGTFDVSLLTIEEGIFEVKATAGDTHLGGEDFDNRMVNHFVQEFKRKNKKDISGNPRALRRLRTACERAKRTLSSTAQTTIEIDSLYEGIDFY 300
  TaHSP70 VLIFDLGGGTFDVSLLTIEEGIFEVKATAGDTHLGGEDFDNRMVNHFVQEFKRKHKKDITGNPRALRRLRTACERAKRTLSSTAQTTIEIDSLYEGVDFY 299
Consensus VLIFDLGGGTFDVSLLTIEEGIFEVKATAGDTHLGGEDFDNRMVNHFVQEFKRK KKDI GNPRALRRLRTACERAKRTLSSTAQTTIEIDSLYEG DFY 285

  NbHSP70 STITRARFEELNMDLFRKCMEPVEKCLRDAKMDKSTIHDVVLVGGSTRIPKVQQLLQDFFNGKELCKSINPDEAVAYGAAVQAAILSGEGNEKVQDLLLL 400
  TaHSP70 TTITRARFEELNMDLFRKCMEPVEKCLRDAKMDKSTVHDVVLVGGSTRIPKVQQLLQDFFNGKELCKSINPDEAVAYGASVQAAILSGEGNEKVQDLLLL 399
Consensus  TITRARFEELNMDLFRKCMEPVEKCLRDAKMDKST HDVVLVGGSTRIPKVQQLLQDFFNGKELCKSINPDEAVAYGA VQAAILSGEGNEKVQDLLLL 382

  NbHSP70 DVTPLSLGLETAGGVMTVLIPRNTTIPTKKEQVFSTYSDNQPGVLIQVYEGERTRTRDNNLLGKFELSGIPPAPRGVPQITVCFDIDANGILNVSAEDKT 500
  TaHSP70 DVTPLSLGLETAGGVMTTLIPRNTTIPTKKEQVFSTYSDNQPGVLIQVYEGERARTKDNNLLGKFELSGIPPAPRGVPQITVCFDIDANGILNVSAEDKT 499
Consensus DVTPLSLGLETAGGVMT LIPRNTTIPTKKEQVFSTYSDNQPGVLIQVYEGER RT DNNLLGKFELSGIPPAPRGVPQITVCFDIDANGILNVSAEDKT 479

  NbHSP70 TGQKNKITITNDKGRLSKEEIEKMVQEAEKYKSEDEEHKKKVEAKNALENYAYNMRNTVKDEKIASKLPEADKKKIEDAIESAIQWLDANQLAESDEFED 600
  TaHSP70 TGQKNKITITNDKGRLSKEEIEKMVQEAERYKAEDEEHKKKVDAKNALENYAYNMRNTVKDDKIASKLGADDKKKVEEAIEGTISWLDANQLAEADEFED 599
Consensus TGQKNKITITNDKGRLSKEEIEKMVQEAE YK EDEEHKKKV AKNALENYAYNMRNTVKD KIASKL   DKKK E AIE  I WLDANQLAE DEFED 566

  NbHSP70 KMKELESICNPIIAKMYQGAGGDMGGAMDDDAPAPSGGSGAGPKIEEVD 649
  TaHSP70 KMKELEGICNPIIAKMYQGAAPDMGGGMGMDEDMPAGGGGAGPKIEEVD 648
Consensus KMKELE ICNPIIAKMYQGA  DMGG M  D   P GG GAGPKIEEVD 604
```

**Figure S3**

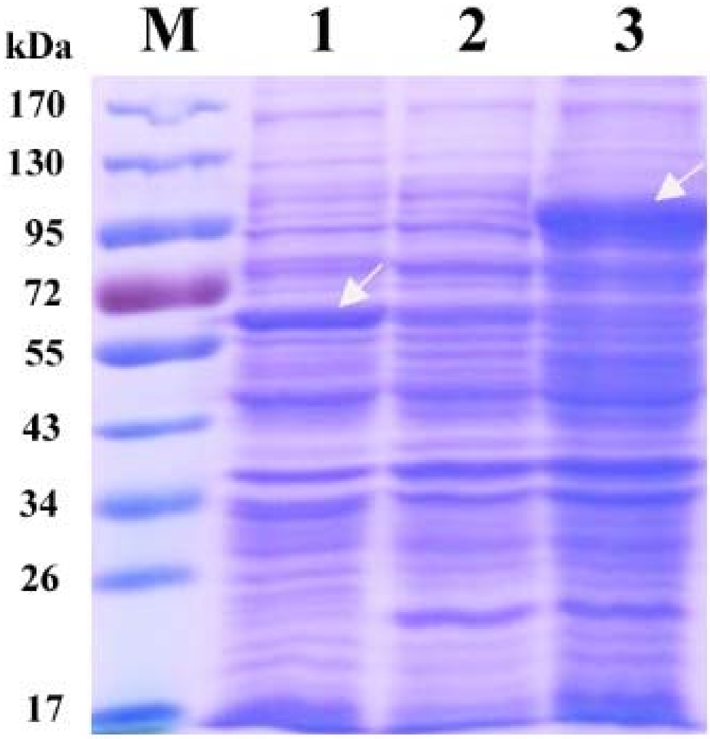

Supplement: Supplementary Tables and Figures [file srep45590-s1.pdf]
